# Usefulness of Combining Sputum and Nasopharyngeal Samples for Viral Detection by Reverse Transcriptase PCR in Adults Hospitalized with Acute Respiratory Illness

**DOI:** 10.1128/spectrum.02775-22

**Published:** 2022-11-14

**Authors:** Keun Ju Kim, Seung Gyu Yun, Yunjung Cho, Myung-Hyun Nam, Chang Kyu Lee

**Affiliations:** a Department of Laboratory Medicine, Korea University Anam Hospital, Seoul, South Korea; b Department of Laboratory Medicine, Korea University College of Medicine, Seoul, South Korea; University of Utah and ARUP Laboratories

**Keywords:** sputum, respiratory virus, combined samples, diagnosis, reverse transcriptase PCR

## Abstract

Nasopharyngeal swabs (NPS) or washings have traditionally been used to diagnose respiratory tract infections. Reverse transcriptase PCR (RT-PCR) is widely used for rapid viral detection using samples from the upper respiratory tract. However, RT-PCR is rarely applied to sputum samples, mainly due to the viscosity of sputum. Thus, we assessed the detection rates of respiratory viruses from NPS, sputum samples, and combined NPS and sputum samples using multiplex RT-PCR (Allplex respiratory panels I, II, and III; Seegene, Seoul, South Korea). Paired NPS and sputum samples were collected from 219 patients admitted to the hospital with acute respiratory illnesses from October to December 2019. RT-PCR was performed on each sample for virus detection. Combined samples for virus detection were produced using remnant NPS and sputum samples with a positive virus signal. Respiratory viral nucleic acid was identified in 92 (42%) of 219 patients. Among the 92 viral detections, 61 (28%) were detected by both NPS and sputum samples. Twenty-four (11%) were sputum positive/NPS negative, and seven (3%) were sputum negative/NPS positive. For the combined NPS-sputum samples (*n* = 92), all paired samples positive in both specimens (*n* = 61) were also positive in the combined NPS-sputum sample. Twenty-seven (87%) of the 31 discordant paired samples were positive in the combined samples. Out of the total of 103 viruses identified before combining the samples, the detection rate of the combined samples was 94% (97/103), which was higher than the detection rates of sputum (88%; 91/103) and NPS (71%; 73/103). Because additional tests incur additional costs, our findings suggest that combining samples instead of testing separate samples using RT-PCR is likely the most cost-effective method of viral testing for patients with acute respiratory illnesses.

**IMPORTANCE** This study reveals that RT-PCR utilizing sputum significantly increased the detection rate for respiratory viral nucleic acids among adult patients admitted to the hospital, compared to nasopharyngeal swabs (NPS). Notably, combined samples of sputum and NPS maintained the majority of the improved sputum detection rate with only a few positive signal losses from NPS samples. In order to detect respiratory viruses in adult patients with acute respiratory illness, it is important to choose the optimal respiratory samples. This study helped to improve our understanding of this process.

## INTRODUCTION

Diagnosis of respiratory viral illnesses is routinely performed by viral culture or antigen testing of specimens from the upper respiratory tract (URT), such as nasopharyngeal swabs (NPS) or washings (1, 2). Although culture-based methods for virus detection have been the gold-standard reference in clinical virology, they are time-consuming and laborious (1). In addition, antigen testing assays are simple, fast, and easy to use; however, the sensitivity is sometimes suboptimal (1). The diagnostic accuracy of respiratory viral infections has been greatly improved with the continuous advancement of molecular amplification methods (1). Sputum was traditionally considered a suboptimal specimen due to its viscosity (2). However, with the improvement of molecular methods, sputum has been introduced for the diagnosis of respiratory viral infections, in addition to samples from the URT (3, 4). Testing of extra samples adds cost. Consequently, combining samples from the URT and sputum into a single tube may be another approach for viral testing. The aim of the current study was to evaluate the detection rates of respiratory viruses in paired NPS and sputum samples and combined samples of NPS and sputum from adult patients admitted with acute respiratory illnesses using multiplex real-time reverse transcriptase PCR (RT-PCR).

## RESULTS

### Increased detection rate in sputum.

Viral nucleic acid was detected in paired samples from 92 of the 219 patients (42%) (Table 1). Of the 92 paired samples, 61 (28%) were positive in both the NPS and sputum samples, 7 (3%) were positive in NPS alone, and 24 (11%) were positive in sputum alone. The positivity rate was significantly higher for sputum (39%; 85/219) than for NPS (31%; 68/219) (*P* = 0.004). In the NPS samples, a single virus was identified in 63 samples (87%) and two viruses in five samples (13%). In sputum, a single virus was detected in 78 samples (89%) and two viruses in seven samples (11%).

**TABLE 1:** Comparison of RT-PCR results for paired and combined NPS and sputum samples^*a*^

Sample type	No. (%) of sample pairs with a positive result	
	Before combining samples	After combining samples
NPS alone	7	6 (86)
Sputum alone	24	21 (88)
Both NPS and sputum	61	61 (100)
Total	92	88 (96)

a NPS, nasopharyngeal swab.

In total, 103 viruses were identified in paired samples from 92 cases (Table 2). The detection rates for any virus were 33% (73/219) for NPS RT-PCR and 42% (91/219) for sputum RT-PCR. Rhinovirus was the most common virus detected, followed by coronavirus OC43 and influenza A virus/subtype pdm09 (Table 2), all of which were more frequently identified in sputum than in NPS.

**TABLE 2:** Distribution of 103 viruses in paired samples from 92 patients with a positive signal^*a*^

Virus	No. (%) of positive results				*P* value^*b*^
	Total	NPS and sputum	NPS only	Sputum only	
Rhinovirus	32	19	2	11	0.005
Coronavirus OC43	17	11	1	5	0.175
Influenza A/pdm09	15	9	1	5	0.169
RSV B	11	9	1	1	1.000
Metapneumovirus	6	5	1	0	
Parainfluenza 1	5	2	1	2	
Adenovirus	4	0	1	3	
Parainfluenza 2	3	2	1	0	
Parainfluenza 4	2	1	1	0	
Enterovirus	2	1	0	1	
RSV A	2	1	0	1	
Coronavirus 229E	1	0	1	0	
HBOV	1	0	0	1	
Influenza A/H3	1	0	1	0	
Total	103	61 (59)	12 (12)	30 (29)	

a NPS, nasopharyngeal swab; RSV, respiratory syncytial virus; HBOV, human bocavirus.

b The chi-square test was used for rhinovirus; Fisher’s exact test was used for coronavirus OC43, influenza A/pdm09, and RSV B.

### Combined nasopharyngeal swab and sputum samples.

**FIG 1:**
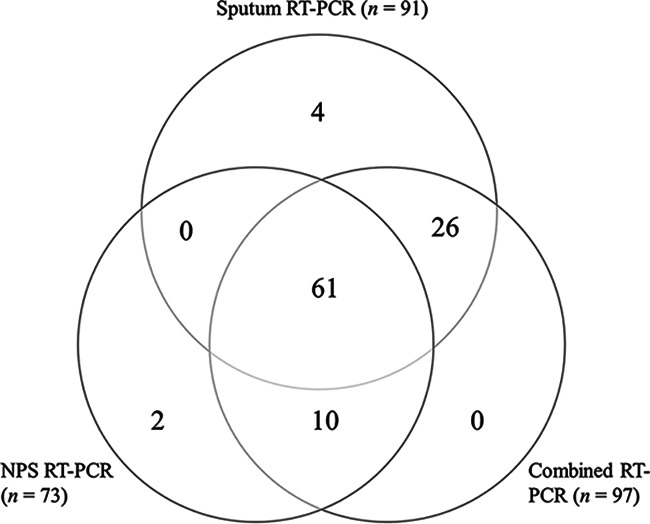
Venn diagram illustrating the distribution and overlap of positive tests for detecting viral nucleic acids. Each section of the diagram shows how many distinct viral nucleic acids were detected by each of the assays (total, *n* = 103).

We combined each of the 92 positive paired samples into a single assay to evaluate the detection rate for a simple combined method. All paired samples that were positive in both NPS and sputum specimens were positive in the combined samples (Table 1). Twenty-seven of the 31 discordant paired samples (87%) were positive by a combined reaction. Overall, 88 of the 92 positive paired samples (96%) were positive in the combined samples. The distribution of positive viral nucleic acid results is shown in Fig. 1. While only two viral signals were lost in the NPS samples after they were combined, the combined samples retained most of the added viral signals of the sputum (26/30). The total virus detection rate for the combined NPS and sputum samples (94%; 97/103) was higher than that of sputum (88%; 91/103) or NPS (71%; 73/103). An overview of all results from the study is presented in Table S1 in the supplemental material.

## DISCUSSION

The development of RT-PCR has revolutionized viral infection diagnostics, and the substantial burden of these etiologic agents is now being increasingly appreciated (3–6). However, important issues still remain regarding choosing the optimal specimens for viral assays (7). With advancements in nucleic acid amplification tests, sputum samples, which are representative of lower respiratory tract secretions, may be used for the diagnosis of respiratory virus infections (2). A few studies have described the increased diagnostic usefulness of sputum compared to specimens from the upper respiratory tract (3, 4, 8–11). Our results again showed that sputum increased the detection rate for respiratory viral nucleic acids. Since some viral nucleic acids were only detected using either sputum or NPS, the testing of both specimens might be the best option to detect respiratory viruses in adult patients who can produce sputum. However, testing of extra samples using RT-PCR adds cost and expenditures (12); therefore, we tried to investigate the usefulness of combining NPS and sputum from the same patient into a single tube for respiratory virus detections. We demonstrated that the increased detection rate for sputum was maintained using combined NPS and sputum samples, which was consistent with another previous study (12). The combined method in the current study was highly sensitive (94%; 97/103), compared to RT-PCR testing of NPS (71%; 73/103) and sputum (88%; 91/103) separately. Only four samples with six viral nucleic acids were not detected using the combined method. Based on the high cycle threshold (*C_T_*) values (>38) for all six missed viruses (38.3, 38.5, 38.7, 39.4, 40.5, and 40.8), a possible reason for missed detection when using the combined method may be sample dilution (13). In principle, combining one positive sample with one negative sample will cause 2-fold viral dilution in the combined sample, which may increase the number of RT-PCR cycles. Thus, false-negative results could occur if viral loads of samples are close to the detection limit, as observed in the current study. Additionally, we stored the samples at −80°C for up to 2 months before combining them, depending on the collection time of each sample. The storage conditions may have partly caused false-negative results, especially for samples with high *C_T_* values (14).

In our study, rhinovirus was more frequently detected in sputum than in NPS (*P* = 0.005), as observed previously (4, 8). The upper respiratory tract was previously known to be the major site for rhinovirus infection. However, introduction of sensitive and technically simple molecular detection systems, especially multiplex RT-PCR, enabled affordable rhinovirus detection in clinical samples (15). Thus, it was discovered that rhinovirus is responsible for a substantial rate of lower respiratory infections in adults (15), which was corroborated by our findings. Human coronavirus OC43 has been associated with both upper and lower respiratory tract infections with various degrees of severity (16, 17). The virus is considered one of the most common human coronaviruses worldwide (17). Our findings showed that the virus was the most frequent coronavirus detected in the current study. The virus was more frequently associated with sputum than NPS. In accordance with previous works (4, 8), we found a higher detection rate of influenza A/pdm09 in sputum than NPS using the RT-PCR assay. This study was performed prior to the coronavirus disease 2019 (COVID-19) pandemic, when rhinovirus, RSV, adenovirus, and influenza virus were most frequently detected in Korea. In addition, the study sample size was small, and the population included only hospitalized patients. Thus, the distribution of viruses detected in our study may be biased based on the dates of sample collection.

The use of sputum as a diagnostic specimen has been underappreciated because of its highly viscous and heterogeneous nature, which makes processing a challenge (4). Additionally, obtaining adequate samples for sputum is more challenging than acquiring NPS samples. Yet our data indicate that the processing difficulty is resolvable with the proper processing method, as in the current study. In addition, the increased detection rate of sputum can be substantial if it can be used for diagnostic assays to detect acute respiratory illnesses. Our study demonstrated that sputum and NPS tests generated complementary results. Thus, the best diagnostic strategy may include performing RT-PCR with both specimens simultaneously for the highest detection rate. However, additional testing incurs additional costs. Due to a clear tendency to shift from fee-for-service payments to a managed-care type of medicine that optimizes efficient use of limited sources, clinical laboratories should maximize testing efficiency and reduce costs (18). Therefore, due to the high retained detection rate in identifying respiratory virus nucleic acids using the combined method, the most cost-effective method may involve combining samples rather than performing separate RT-PCR tests. However, there will be an increase in the sample processing time and hands-on personnel time, which might offset some of the savings made by combining samples.

Our study has several limitations. First, this study was limited to the small sample size of the adult patients who could produce sputum at a single center. Accordingly, our findings may not be generalized to represent other study groups, such as children or very sick patients on respiratory support who cannot generate sputum in other centers. Further studies with a different and large study population at other centers are needed to validate our findings. Second, as previously stated, we cannot exclude the possibility that the effect of storing samples before combining them might have resulted in loss of the positive signals. The detection rate of the combined method might have been higher with timely combination of the samples. Third, this study was performed prior to the emergence of severe acute respiratory syndrome coronavirus 2 (SARS-CoV-2) and the COVID-19 pandemic. It would be interesting to perform future studies on hospitalized patients in the SARS-CoV-2 era.

In conclusion, we showed that using sputum specimens with RT-PCR significantly increased the detection rate for respiratory viral nucleic acids among adult patients admitted to the hospital. More importantly, combining samples of NPS and sputum maintained the added detection rate of the sputum. Therefore, the most efficient and cost-effective method may be combining samples instead of performing RT-PCR on separate samples.

## MATERIALS AND METHODS

### Study population.

Adults over 21 years of age admitted to Korea University Anam Hospital with symptoms consistent with acute respiratory infections (cough, sore throat, dyspnea, nasal congestion, sputum, myalgia, or fever) were recruited from 1 October 2019 to 30 December 2019. Nonduplicate paired specimens of NPS and sputum were obtained from 219 patients. This study was approved by the institutional review board (IRB) of Korea University Anam Hospital (2019AN0542). A waiver of informed consent was granted by the IRB.

### Nasopharyngeal swabs.

NPS samples were collected from each patient using flocked swabs (Copan Diagnostics; Brescia, Italy) and placed into a tube with 3 mL universal transport medium (UTM; Copan Diagnostics). Each UTM tube contained three glass beads, which facilitated the release and dispersion of patient materials and virus particles during vortexing. After vortexing each tube, 2 mL UTM was transferred to a 2-mL tube and centrifuged at 13,000 × *g* for 1 min. The supernatants were used for nucleic acid extraction. Residual samples were stored at −80°C until combination for further testing.

### Sputum.

The expectorated sputum specimens from each patient were collected in sterile containers and delivered to the clinical laboratory as soon as possible. The sputum specimens were then diluted with an equal volume of phosphate-buffered saline and liquefied by vortexing with the aid of glass beads. Vortexing with glass beads facilitated the release of any cell-associated virus particles from the patient materials. A 2-mL aliquot of each liquefied sputum specimen was transferred and centrifuged as described for NPS. The supernatants were used for further RT-PCR testing. The sample remnants were stored at −80°C.

### Nucleic acid extraction.

Nucleic acid was extracted from 300 μL of the NPS and sputum samples using the Microlab STARlet IVD (Hamilton Robotics, Reno, NV) with the STARMag 96 × 4 universal cartridge kit (Seegene, Seoul, South Korea). Using the automated liquid handling workstation, the nucleic acid extraction and PCR setup was implemented. The final elution volume of each extracted nucleic acid was 100 μL. An exogenous internal control was added to the sample prior to extraction not only to check nucleic acid extraction but also to identify any RT-PCR inhibition.

### Real-time RT-PCR detection assay.

The multiplex real-time RT-PCR detection assay was performed using the Allplex respiratory panel I, II, and III detection kits (Seegene) according to the manufacturer’s instructions. NPS and sputum samples from each patient were tested in parallel for the following 19 respiratory viruses: influenza A virus, influenza B virus, human respiratory syncytial virus A, human respiratory syncytial virus B, and subtyping of influenza A virus (human influenza A virus subtypes H1, H3, and H1pdm09) for panel I; human adenovirus, human metapneumovirus, human enterovirus, human parainfluenza virus 1, human parainfluenza virus 2, human parainfluenza virus 3, and human parainfluenza virus 4 for panel II; and human bocavirus 1/2/3/4, human rhinovirus, human coronavirus 229E, human coronavirus NL63, and human coronavirus OC43 for panel III. A cycle threshold (*C_T_*) value of ≤42 was considered positive for any virus detection. These tests have not been validated for any other specimens than NPS, nasopharyngeal aspirate, and bronchial lavage.

### Combined sputum and nasopharyngeal swab samples.

After performing the RT-PCR test on the paired NPS and sputum samples from patients, the paired samples that were NPS positive only, sputum positive only, or both NPS and sputum positive were selected for further combined analysis. The residual paired samples stored at −80°C were thawed and centrifuged at 13,000 × *g* for 1 min. The remnants of the paired samples were combined into a new single 2-mL tube by transferring a 1-mL aliquot of the supernatant of each specimen using a pipette and mixed by vortexing. Nucleic acid extraction of the combined samples and RT-PCR for the 19 viruses were conducted as described above.

### Statistical analysis.

The McNemar test was used to determine the difference between the positivity rates of the paired NPS and sputum samples. The detection rate difference in any virus for each specimen type was analyzed using the chi-square test or Fisher’s exact test. All statistical analyses were performed using SPSS for Windows v26.0 (IBM, Armonk, NY, USA). A *P* value of <0.05 was considered statistically significant.

## References

[1] Gradisteanu Pircalabioru G, Iliescu FS, Mihaescu G, Cucu AI, Ionescu ON, Popescu M, Simion M, Burlibasa L, Tica M, Chifiriuc MC, Iliescu C. 2022. Advances in the rapid diagnostic of viral respiratory tract infections. Front Cell Infect Microbiol 12:807253. doi:10.3389/fcimb.2022.807253. 35252028 PMC8895598

[2] Dunn JJ, Pinsky BA. 2019. Specimen collection, transport, and processing: virology, p 1455–1456. *In* Carroll KC, Pfaller MA, Landry ML, McAdam AJ, Patel R, Richter SS, Warnock DW (ed), Manual of clinical microbiology, 12th ed. ASM Press, Washington, DC.

[3] Falsey AR, Formica MA, Walsh EE. 2012. Yield of sputum for viral detection by reverse transcriptase PCR in adults hospitalized with respiratory illness. J Clin Microbiol 50:21–24. doi:10.1128/JCM.05841-11. 22090400 PMC3256730

[4] Jeong JH, Kim KH, Jeong SH, Park JW, Lee SM, Seo YH. 2014. Comparison of sputum and nasopharyngeal swabs for detection of respiratory viruses. J Med Virol 86:2122–2127. doi:10.1002/jmv.23937. 24797344 PMC7166652

[5] Beckham JD, Cadena A, Lin J, Piedra PA, Glezen WP, Greenberg SB, Atmar RL. 2005. Respiratory viral infections in patients with chronic, obstructive pulmonary disease. J Infect 50:322–330. doi:10.1016/j.jinf.2004.07.011. 15845430 PMC7132437

[6] Caram LB, Chen J, Taggart EW, Hillyard DR, She R, Polage CR, Twersky J, Schmader K, Petti CA, Woods CW. 2009. Respiratory syncytial virus outbreak in a long-term care facility detected using reverse transcriptase polymerase chain reaction: an argument for real-time detection methods. J Am Geriatr Soc 57:482–485. doi:10.1111/j.1532-5415.2008.02153.x. 19187415 PMC7166908

[7] Singh K, Vasoo S, Stevens J, Schreckenberger P, Trenholme G. 2010. Pitfalls in diagnosis of pandemic (novel) A/H1N1 2009 influenza. J Clin Microbiol 48:1501–1503. doi:10.1128/JCM.02483-09. 20164266 PMC2849562

[8] Branche AR, Walsh EE, Formica MA, Falsey AR. 2014. Detection of respiratory viruses in sputum from adults by use of automated multiplex PCR. J Clin Microbiol 52:3590–3596. doi:10.1128/JCM.01523-14. 25056335 PMC4187748

[9] Lin C, Xiang J, Yan M, Li H, Huang S, Shen C. 2020. Comparison of throat swabs and sputum specimens for viral nucleic acid detection in 52 cases of novel coronavirus (SARS-Cov-2)-infected pneumonia (COVID-19). Clin Chem Lab Med 58:1089–1094. doi:10.1515/cclm-2020-0187. 32301745

[10] Wang L, Yang S, Yan X, Liu T, Feng Z, Li G. 2019. Comparing the yield of oropharyngeal swabs and sputum for detection of 11 common pathogens in hospitalized children with lower respiratory tract infection. Virol J 16:84. doi:10.1186/s12985-019-1177-x. 31234918 PMC6591818

[11] Nyawanda BO, Njuguna HN, Onyango CO, Makokha C, Lidechi S, Fields B, Winchell JM, Katieno JS, Nyaundi J, Ade F, Emukule GO, Mott JA, Otieno N, Widdowson MA, Chaves SS. 2019. Comparison of respiratory pathogen yields from nasopharyngeal/oropharyngeal swabs and sputum specimens collected from hospitalized adults in rural western Kenya. Sci Rep 9:11237. doi:10.1038/s41598-019-47713-4. 31375774 PMC6677726

[12] Falsey AR, Formica MA, Walsh EE. 2012. Simple method for combining sputum and nasal samples for virus detection by reverse transcriptase PCR. J Clin Microbiol 50:2835. doi:10.1128/JCM.01473-12. 22692748 PMC3421505

[13] Bateman AC, Mueller S, Guenther K, Shult P. 2021. Assessing the dilution effect of specimen pooling on the sensitivity of SARS-CoV-2 PCR tests. J Med Virol 93:1568–1572. doi:10.1002/jmv.26519. 32936471

[14] Yilmaz Gulec E, Cesur NP, Yesilyurt Fazlioğlu G, Kazezoğlu C. 2021. Effect of different storage conditions on COVID-19 RT-PCR results. J Med Virol 93:6575–6581. doi:10.1002/jmv.27204. 34260086 PMC8426709

[15] Civljak R, Tot T, Falsey AR, Huljev E, Vranes J, Ljubin-Sternak S. 2019. Viral pathogens associated with acute respiratory illness in hospitalized adults and elderly from Zagreb, Croatia, 2016 to 2018. J Med Virol 91:1202–1209. doi:10.1002/jmv.25437. 30801727 PMC7166480

[16] Vabret A, Mourez T, Gouarin S, Petitjean J, Freymuth F. 2003. An outbreak of coronavirus OC43 respiratory infection in Normandy, France. Clin Infect Dis 36:985–989. doi:10.1086/374222. 12684910 PMC7109673

[17] Gaunt ER, Hardie A, Claas EC, Simmonds P, Templeton KE. 2010. Epidemiology and clinical presentations of the four human coronaviruses 229E, HKU1, NL63, and OC43 detected over 3 years using a novel multiplex real-time PCR method. J Clin Microbiol 48:2940–2947. doi:10.1128/JCM.00636-10. 20554810 PMC2916580

[18] Samuel LP, Hansen GT, Kraft CS, Pritt BS, ASM Clinical and Public Health Microbiology Committee. 2021. The need for dedicated microbiology leadership in the clinical microbiology laboratory. J Clin Microbiol 59:e0154919. doi:10.1128/JCM.01549-19. 33597258 PMC8288296

