# Supplementary material for: Usefulness of Combining Sputum and Nasopharyngeal Samples for Viral Detection by Reverse Transcriptase PCR in Adults Hospitalized with Acute Respiratory Illness
Source: Microbiol Spectr. 2022 Nov 14;10(6):e02775-22. doi: 10.1128/spectrum.02775-22 (PMC9769557; doi:10.1128/spectrum.02775-22)
Supplement: Supplemental file 1 — Table S1. Download spectrum.02775-22-s0001.pdf, PDF file, 0.06 MB [file spectrum.02775-22-s0001.pdf]

Table S1 Overview of comparison results for paired NPS and sputum samples and combined positive NPS and sputum samples

Type of sample	No. sample pairs tested before combining	No. sample pairs (%) with a positive result after combining	No. virus detected before combining	No. virus detected (%) after combining
NPS[a] positive alone	7	6 (86)	12	10 (83)
Sputum positive alone	24	21 (88)	30	26 (87)
Both NPS and sputum positive	61	61 (100)	61	61 (100)
Total number of positives	92	88 (96)	103	97 (94)

Virus	No. of positive viral nucleic acid detection before combining				No. of positive viral nucleic acid detection after combining			
	Total	NPS and sputum	NPS only	Sputum only	Total	NPS and sputum	NPS only	Sputum only
Rhinovirus	32	19	2	11	29	19	2	8[b]
Coronavirus OC43	17	11	1	5	16	11	1	4[c]
Influenza A/pdm09	15	9	1	5	15	9	1	5

RSV B	11	9	1	1		11	9	1	1
Metapneumovirus	6	5	1	0		6	5	1	0
Parainfluenza 1	5	2	1	2		4	2	0[d]	2
Adenovirus	4	0	1	3		3	0	0[e]	3
Parainfluenza 2	3	2	1	0		3	2	1	0
Parainfluenza 4	2	1	1	0	-	2	1	1	0
Enterovirus	2	1	0	1	-	2	1	0	1
RSV A	2	1	0	1	-	2	1	0	1
Coronavirus 229E	1	0	1	0	-	1	0	1	0
HBOV	1	0	0	1	-	1	0	0	1
Influenza A/H3	1	0	1	0	-	1	0	1	0
Total	103	61	12	30		97	61	10	26

[a]NPS, nasopharyngeal swab; RSV, respiratory syncytial virus; HBOV, human bocavirus.

[b]Ct value prior to combining for each of the three not detected viruses was 38.7, 39.4, and 40.5.

[C]Ct value prior to combining for the not detected virus was 38.5.

[d]Ct value prior to combining for the not detected virus was 38.3.

[e]Ct value prior to combining for the not detected virus was 40.8.
